# Brain sparing in fetal growth restriction: The double‐edged sword of fetal hypoxaemia

**DOI:** 10.1113/JP291253

**Published:** 2026-07-06

**Authors:** Beth J. Allison, Tegan A. White, Emily J. Camm, Suzanne L. Miller

**Affiliations:** ^1^ The Ritchie Centre, Department of Obstetrics and Gynaecology Monash University Clayton Victoria Australia; ^2^ Hudson Institute of Medical Research Clayton Victoria Australia

**Keywords:** brain sparing, cardiovascular, fetal growth restriction, hypoxia, neuropathology, small for gestational age

## Abstract

Fetal hypoxaemia during pregnancy is not uncommon, arising from chronic placental insufficiency or acute stressors. In the case of placental insufficiency and chronic fetal hypoxia, fetal growth is reduced, resulting in fetal growth restriction (FGR). Fetal hypoxaemia initiates an immediate adaptive strategy to preserve brain oxygen delivery – the brain sparing response. Asymmetric FGR, in which head size is relatively larger than body size, is evidence of prolonged brain sparing. The acute physiology of the brain sparing response is well‐defined in preclinical studies, involving peripheral vasoconstriction and reduced cerebral resistance to promote cerebral vasodilatation. Yet, the mechanisms that maintain fetal brain sparing during sustained hypoxaemia remain incompletely understood. Furthermore, although brain sparing has historically been interpreted as protective, this concept is being challenged, with its presence linked to increased risk of death or neonatal morbidity and neuropathology. This contradiction reflects that brain sparing is not static but evolves with the severity of fetal hypoxaemia; clinical evidence points to a front‐to‐back pattern of brain vasodilatation that initially prioritises cortical perfusion for higher‐order function, whereas prolonged or severe hypoxaemia drives a shift towards brainstem preservation and survival. Thus, the brain sparing response is initiated by fetal hypoxaemia and is a unique indicator of fetal compromise, but it progresses from a compensatory to a maladaptive response. The mechanisms, multisystem physiology and ontogeny of sustained brain sparing in severe FGR are not well characterised, but advancing this knowledge affords new opportunities to diagnose and manage FGR, and to intervene to prevent adverse consequences.

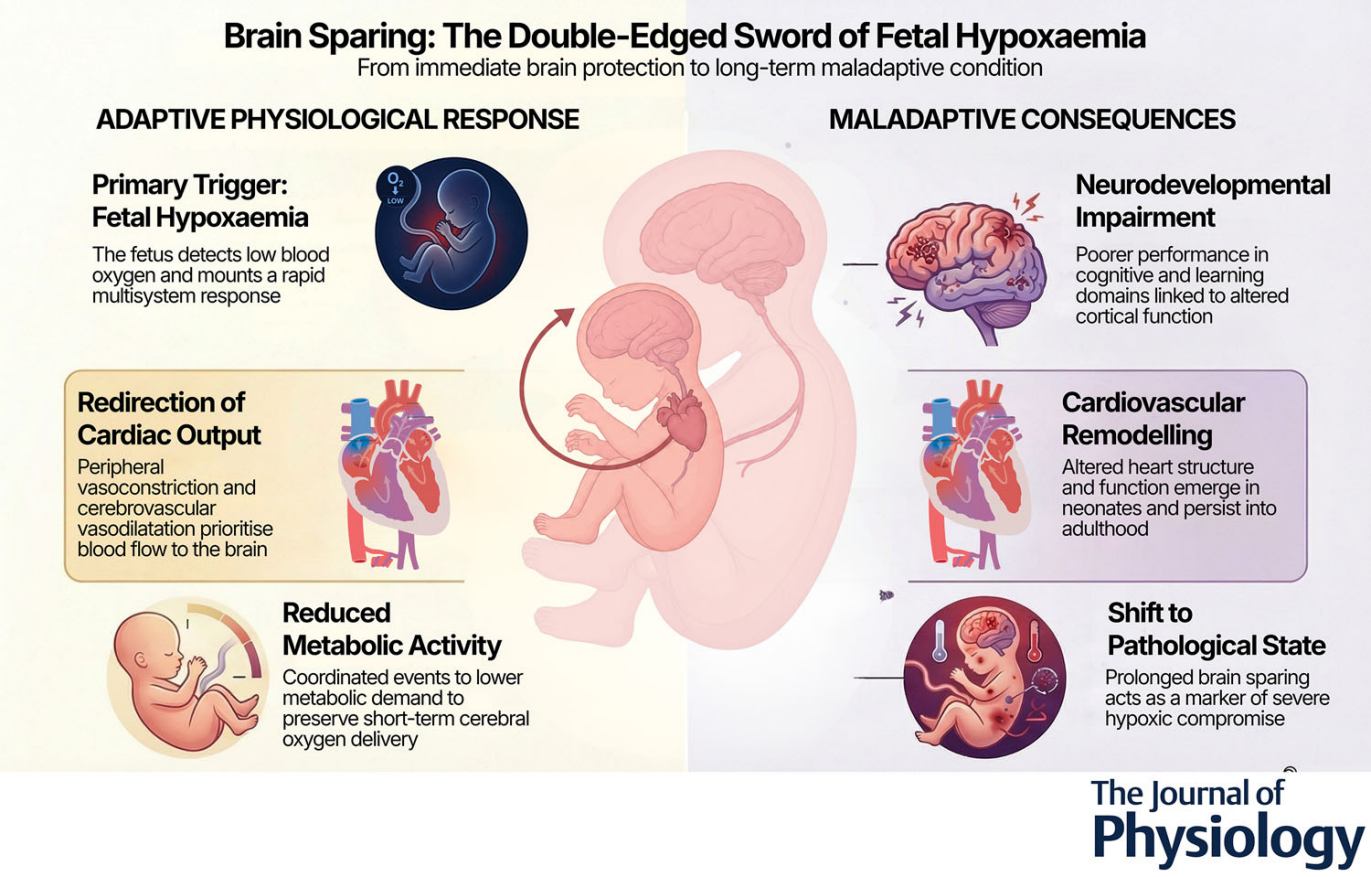

## Graphical abstract

**Abstract figure legend** The double‐edged sword of brain sparing. Fetal hypoxaemia triggers a coordinated adaptive response characterised by redistribution of cardiac output toward essential organs, including the brain, and a reduction in overall metabolic activity to preserve oxygen supply. While these mechanisms provide immediate neuroprotection, prolonged or severe hypoxaemia leads to maladaptive consequences. These include neurodevelopmental impairment, adverse cardiovascular remodelling, and a transition from physiological adaptation to a pathological state, highlighting brain sparing as a double‐edged sword.

## Introduction

It has long been recognised that the fetus develops in a relatively low‐oxygen environment – Sir Joseph Barcroft coined the phrase ‘Everest *in utero*’ (Giussani et al., 2016). This is true in the sense that the partial pressure of oxygen is low in the fetus relative to the adult; however, this is physically normal for the *in utero* environment, with oxygen delivery matched to meet fetal metabolic demands and to support growth. Fetal compromise emerges when this balance is disrupted and oxygen supply to the fetus is reduced, causing low fetal arterial blood oxygen concentration (hypoxaemia).

Fetal and placental adaptations are present from early pregnancy to ensure delivery of oxygen to tissues despite a low gaseous diffusing potential, including a relatively high fetal cardiac output, vasodilatation of the placental and fetal vasculature, and a high capacity for oxygen carrying in the fetal blood (Giussani & Davidge, 2013). These mechanisms maintain adequate oxygen delivery to the fetus under normal physiological conditions. However, clinically significant reductions in fetal oxygen supply are relatively common in pregnancy, arising from a chronic challenge as occurs with placental insufficiency, but also during labour when uterine contractions can transiently reduce fetal oxygenation. Placental insufficiency reduces oxygen transfer to the fetus and is the primary cause of fetal growth restriction (FGR). Consistent with chronic placental compromise, cord blood from FGR and small for gestational age (SGA) human infants show lower oxygenation (reduced partial pressure of oxygen and oxygen saturation) compared to appropriately grown infants, alongside differences in acid–base balance and substrate availability (Cetin et al., 2020; Lackman et al., 2001; Nicolaides et al., 1989). Together, these findings indicate that placental compromise causes fetal hypoxaemia and reduces substrate availability, resulting in a compensatory decrease in fetal metabolic rate, decreasing both oxygen and glucose consumption normalised for body weight (Cetin et al., 2020). Over time, this adaptive response constrains somatic growth and contributes to the development of FGR (Dall'Asta et al., 2023; MacDonald et al., 2019).

The fetus mounts an acute physiological response to hypoxaemia, increasing cerebral perfusion by redistributing cardiac output towards the brain at the expense of non‐essential organs – this response is termed *brain sparing* (Cohn et al., 1974; Giussani et al., 1993). The brain sparing response is an adaptive mechanism to maintain cerebral homeostasis when oxygen availability is reduced. When prolonged, brain sparing preferentially protects brain growth relative to other organs, resulting in asymmetric FGR (head size is increased relative to other organs) (Giussani, 2016; Miller et al., 2016; Pearce, 2006). Despite this apparent protection, there is now a growing body of evidence linking the presence of brain sparing with neurodevelopmental deficits (Benitez‐Marin et al., 2021; Meher et al., 2015). This perceived contradiction between a sparing effect on brain growth and an association with neurodevelopmental impairments raises important questions regarding whether brain sparing shifts from compensatory to a maladaptive response, which fetuses are most vulnerable, and how the duration and severity of fetal hypoxaemia influence outcomes. Clinically, the persistence and worsening of placental insufficiency and fetal deterioration can be tracked using Doppler ultrasound (Baschat et al., 2001), yet Doppler abnormalities do not capture tissue perfusion or predict individual trajectories. Earlier work has elegantly synthesised the fetal brain sparing response as an acute, reflex redistribution of cardiac output during hypoxia (reviewed in Giussani, 2016), the integrative cardiovascular physiology (Jensen & Berger, 1991) or the neurodevelopmental correlates of brain sparing in FGR (Miller et al., 2016). This Topical Review brings together experimental and clinical evidence to consider what is known, and unknown, regarding the brain sparing response in cases of chronic hypoxaemia. Here we examine the physiology underpinning the acute and sustained brain sparing response, how it is detected in clinical practice, and the postnatal and longer‐term consequences of fetal brain sparing associated with fetal growth restriction.

## Fetal growth restriction

Brain sparing is most often observed as part of the fetal response to placental insufficiency and the broader phenotype of FGR. Defining FGR and distinguishing pathological FGR from constitutionally SGA infants provides essential context for interpreting brain sparing physiology and its prognostic significance. In the past, FGR, or its predecessor term *intrauterine growth restriction* (IUGR), was used interchangeably with SGA to indicate a high‐risk small infant (Bendix et al., 2020). However, SGA babies are not necessarily hypoxaemic or poorly grown, and there is now a consensus that SGA more appropriately reflects infants with an estimated fetal weight (or birth weight) less than the 10th percentile on reference growth charts for gestation and sex (Gordijn et al., 2016; Lees et al., 2020). Using this definition, SGA includes many infants with FGR but also infants who are small but healthy. A diagnosis of true FGR requires measures of fetal biometry, utero‐placental Doppler, and/or fetal growth velocity (Sovio et al., 2015). Most commonly, FGR is defined as estimated fetal weight or abdominal circumference less than the 10th percentile on reference charts together with abnormal antenatal utero‐placental Dopplers, or estimated fetal weight or abdominal circumference less than the third percentile as a stand‐alone parameter (Gordijn et al., 2016). When these definitions are applied, the contemporary rate of FGR in high‐resource birth settings is ∼7% of pregnancies (Liu et al., 2014; Ormesher et al., 2020; Piscopo et al., 2025). As expected, the rate of SGA is higher, by definition affecting about 10% of pregnancies (with reported ranges between 7.7% and 19%) (Cowan et al., 2021; Gardosi et al., 2013; He et al., 2021; Ormesher et al., 2020; Piscopo et al., 2025). SGA remains a common proxy for FGR when antenatal ultrasound evaluation of uteroplacental function is not available, reflecting that SGA infants (<10th percentile) also have an elevated risk for perinatal mortality or morbidity compared to appropriately grown infants (Lees et al., 2020).

FGR is further classified as either early‐onset FGR when diagnosed at <32 weeks’ gestation, or late‐onset FGR at >32 weeks (Figueras & Gratacos, 2014). The distinction between early‐ and late‐onset reflects the pathogenesis of placental insufficiency, with early‐onset FGR usually corresponding to the most severe and/or prolonged placental deficits, present in 20–30% of FGR cases (Figueras & Gratacos, 2014). In both early‐ and late‐onset FGR, brain sparing is described when the baby's head or brain size is relatively normal, while the rest of the body is smaller than expected, measured as an elevated ratio of head circumference or brain volume to abdominal circumference or liver volume (Damodaram et al., 2012; Geva et al., 2006). Thus, asymmetric FGR is characterised by a normal head size but shorter and/or thinner body size at birth, reflecting that *in utero* fetal hypoxia has caused a sustained haemodynamic shift favouring fetal cardiac output towards the brain at the expense of other organ development.

## Normal arterial blood supply in the developing brain

**Figure 1:**
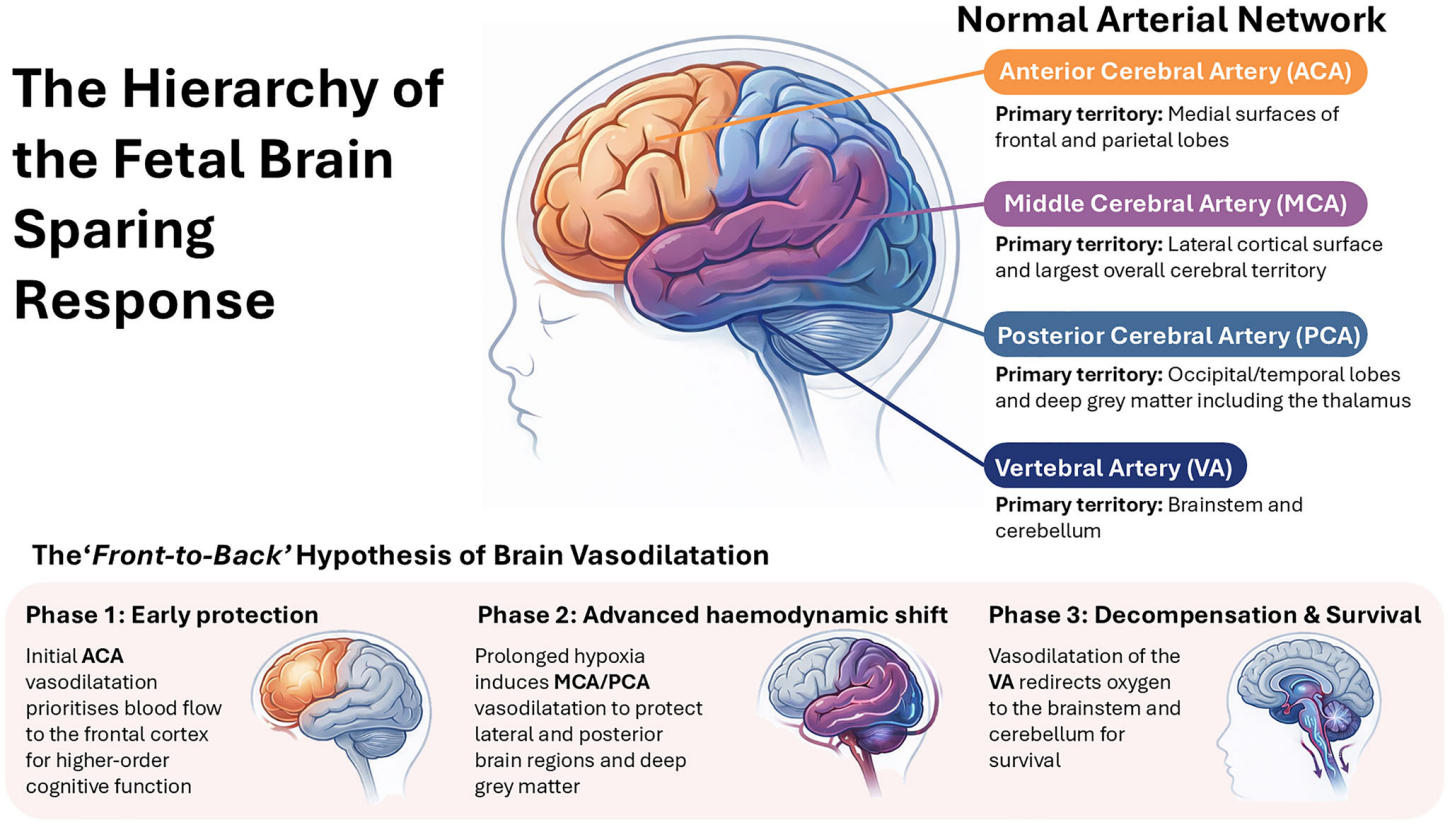
The hierarchy of the fetal brain sparing response The normal network of arterial supply to the brain involves the anterior cerebral artery (ACA), middle cerebral artery (MCA), posterior cerebral artery (PCA) and the vertebral artery (VA). In response to chronic fetal hypoxaemia, as occurs with FGR, there is evidence of a ‘front‐to‐back’ hierarchy of arterial vasodilatation which preferentially shunts oxygenated blood to critical brain regions.

The adult and developing fetal brain is supplied by four principal arteries, namely the paired internal carotid arteries (anterior circulation) and the vertebral–basilar arteries (posterior circulation), which join at the base of the brain via the circle of Willis (Tan & Roberts, 2021). The cerebral vasculature begins forming in early embryogenesis, with the internal carotid artery emerging as a dominant early supplier of the primitive brain and the posterior circulation developing in parallel as the hindbrain enlarges (Kathuria et al., 2011). By late embryonic stage, the circle of Willis is formed. This developmental framework gives rise to the organisation of cerebral blood supply in which the internal carotid system constitutes the anterior circulation and accounts for between 76% and 82% of cerebral blood flow (Boyajian et al., 1995; Scheel et al., 2000), while the vertebral–basilar system supplies the posterior circulation (Boyajian et al., 1995). The paired (left and right) middle cerebral artery (MCA) is the larger of the two main arteries branching from the internal carotid, the other being the paired anterior cerebral artery (ACA). The ACA courses forward and medially to provide blood supply to the frontal lobe and medial aspect of the parietal lobe. The MCA extends laterally towards the cortical surface of the brain and is the primary blood supply to the cortex, with the exception of the ACA supply noted above, and the temporal and occipital lobes are supplied by the posterior cerebral artery. The MCA also supplies the internal capsule and basal ganglia brain regions (Purves et al., 2001). The posterior circulation originates from paired vertebral arteries (VAs), which ascend through the neck and merge at the level of the pons to form a single basilar artery. The basilar artery terminates by giving rise to the paired posterior cerebral artery (PCA), supplying the occipital lobe and deep brain structures including the midbrain, thalamus and hippocampus (Fig. 1). The PCA is commonly described across four segments (P1–P4) and connects with the anterior circulation via the circle of Willis. Branches of the basilar artery also supply the upper spinal cord, brainstem and cerebellum (Purves et al., 2001). This provides a high‐level description of the major arteries and arterial territories of the brain, with multiple anastomoses operating within the brain, and many brain regions served by multiple sources; for example, the hippocampus is primarily served by branches of the PCA with varying contributions from the anterior choroidal artery (distal branches of the internal carotid artery), which are differentially regulated under conditions of hypoxia (Johnson, 2023). Overall, the MCAs are the largest cerebral arteries and supply blood across the largest cerebral territory, including the lateral hemispheres; the ACAs predominantly serve frontal–medial brain regions, and the PCAs supply blood to the posterior cortex, deep grey matter and brainstem (Purves et al., 2001) (Fig. 1). Clinically, these vessels are most commonly interrogated using fetal Doppler ultrasound. From the end of the second trimester of healthy pregnancy (∼28–30 weeks), there is a significant decline in Doppler pulsatility index values across the main fetal cerebral vessels, the MCA, ACA and PCA, reflecting a normal pattern of brain growth and increasing cerebral vasodilatation (Benavides‐Serralde et al., 2010; Ebbing et al., 2007; Morales‐Rosello et al., 2015).

## Detecting placental insufficiency and brain sparing in human FGR

Monitoring placental function and fetal growth throughout pregnancy is a key component of antenatal care, enabling assessment of fetal wellbeing and guiding clinical management. Placental insufficiency and impaired fetal growth are major contributors to perinatal mortality and morbidity (Malhotra et al., 2019), underscoring the importance of timely recognition and surveillance. Delivery is indicated when placental function and/or fetal condition becomes significantly compromised, since FGR is the largest risk factor for stillbirth (Gardosi et al., 2013). Fetal biophysical parameters are assessed during pregnancy via ultrasound, including estimated fetal weight (EFW), abdominal circumference (AC), head circumference (HC), biparietal diameter and femur length (FL), but these are only meaningful when performed serially in a fetus, or when compared to customised growth charts (Lees et al., 2020). Ratios of HC/AC or FL/HC can be used to differentiate between symmetrical and asymmetrical growth restriction with an indication of brain sparing, and when used serially can track the profile of growth and wellbeing over time (Melamed et al., 2021).

Biophysical measures of fetal growth together with utero‐placental Doppler velocimetry provide functional information about placental and fetal wellbeing (Soothill et al., 1993). The Doppler pulsatility index (PI) is used to assess flow dynamics within a vessel and is calculated as the peak systolic velocity minus end diastolic velocity divided by mean velocity. PI provides a reliable non‐invasive measure of vascular resistance; a higher PI reflects greater impedance to normal blood flow, consistent with increased vascular resistance, and in turn, reduced tissue perfusion (Lees et al., 2020). Increased utero‐placental impedance together with reduced cerebral vascular impedance are key determinants of fetal brain sparing (Lees et al., 2020). Elevated uterine artery PI (high resistance blood flow to the placenta) and elevated PI in umbilical arteries (UA) (high resistance from the placenta) directly reflect placental dysfunction (Lees et al., 2022). For this reason, umbilical artery Doppler is universally recommended in the management of FGR, as it can be used to monitor the haemodynamics associated with placental dysfunction (Melamed et al., 2021). UA‐PI >2 standard deviations above the mean is considered an early indicator of abnormal UA blood with elevated placental resistance, which may deteriorate to reversed or absent UA flow indicative of severe placental dysfunction (Figueroa‐Diesel et al., 2007; Lees et al., 2020; Turan et al., 2008). The first Doppler abnormality observed with placental insufficiency is usually an elevated UA‐PI (Turan et al., 2008). Consistent with fetal haemodynamic adaptation to placental compromise, MCA vasodilatation is present in more than 80% of fetuses with an increased UA‐PI, indicative of a fetal brain sparing response to placental dysfunction (Benavides‐Serralde et al., 2014).

The MCA is the largest branch of the internal carotid artery and its superficial position in the brain makes it the most routinely examined fetal cerebral artery on ultrasound to assess cerebral blood flow. In response to placental insufficiency and fetal hypoxaemia, the fetus reduces impedance in the cerebral arteries, causing vasodilatation and increasing brain blood flow perfusion (Figueroa‐Diesel et al., 2007). In clinical practice, vasodilatation of the MCA (reduced MCA‐PI), is the primary measure of brain vasodilatation, reported as MCA‐PI less than the fifth or less than the 10th percentile, or MCA‐PI more than 2 standard deviations below the gestational age‐related mean (Figueras & Gratacos, 2017; Hernandez‐Andrade et al., 2012; Lees et al., 2020; Meher et al., 2015). Since the early 1990s, studies have reported an association between reduced MCA pulsatility and the presence of fetal hypoxaemia (cord blood $P_{O_{2}}$) in SGA/FGR infants, directly linking fetal hypoxaemia with the brain sparing response (Vyas et al., 1990). However, direct assessments of fetal hypoxaemia in human pregnancies are not routinely available, and therefore Doppler measures that integrate increased placental resistance with cerebral adaptation are employed. The cerebroplacental ratio (CPR) represents the ratio of the PI in the MCA relative to the umbilical artery (UA)(MCA‐PI/UA‐PI). By combining fetal cerebral and placental resistance, CPR provides a greater sensitivity than either measure alone for identifying fetal haemodynamic adaptation: a low CPR (<5^th^ percentile, <2 standard deviations below gestation‐matched values, or ratio <1) indicates blood flow redistribution towards the fetal brain as occurs with placental insufficiency and FGR (Flood et al., 2014; Hernandez‐Andrade et al., 2013; Kennedy et al., 2020; Turan et al., 2008). A reduced CPR can provide early evidence of the fetal haemodynamic response to placental dysfunction (Turan et al., 2008), and serial ultrasound assessments show that a reduced fetal growth velocity in the second half of pregnancy is associated with a low CPR (Kennedy et al., 2020). Some studies report the umbilico‐cerebral ratio (UCR), which is simply the inverse of CPR, using the ratio of UA‐PI/MCA‐PI (Coenen et al., 2022). Abnormal Doppler findings in the fetal ductus venosus (DV) are less commonly reported, but are clinically important: an elevated DV‐PI and/or absent or reversed A‐wave flow is typically considered a progressive late‐stage indicator of fetal demise and a prompt for immediate delivery of the fetus (Frusca et al., 2018; Turan et al., 2008).

## The profile of cerebral artery vasodilatation in human FGR

Vasodilatation of the cerebral circulation in response to sustained reduction in oxygen availability is dynamic and regional within the brain, rather than global, with clinical studies introducing the front‐to‐back hypothesis of cerebral artery vasodilatation (Fig. 1) (Steller et al., 2022). In growth restricted fetuses, Doppler studies demonstrate that the three major cerebral arteries show vessel‐specific changes in pulsatility over time as utero‐placental function worsens, with differing trajectories across the ACA, MCA and PCA (Figueroa‐Diesel et al., 2007; Steller et al., 2022) rather than a single global cerebral response.

Sequence studies indicate that Doppler evidence of placental dysfunction, most commonly an elevated UA‐PI, precedes cerebral vasodilatation and acts as the catalyst for fetal haemodynamic adaptation (Turan et al., 2008). Within the cerebral circulation, an early reduction in ACA‐PI is observed which is maintained as hypoxaemia becomes prolonged or worsens, often occurring before detection of MCA vasodilatation (Figueroa‐Diesel et al., 2007; Hernandez‐Andrade et al., 2012; Rossi et al., 2011; Steller et al., 2022). Although the MCA is the most commonly assessed vessel, longitudinal data indicate that the MCA shows a limited early vasodilator response to fetal hypoxia and responds with advancing compromise (Figueroa‐Diesel et al., 2007), supporting the use of reduced MCA‐PI as a marker of established brain sparing in response to significant placental dysfunction (Figueroa‐Diesel et al., 2007; Rossi et al., 2011). Vasodilatation of the PCA tends to follow the profile of ACA dilatation, occurring in advance of MCA changes, but also with a high correlation between PCA and MCA vasodilatation (Benavides‐Serralde et al., 2014; Steller et al., 2022). In severe or prolonged compromise, cerebral vasodilatation may no longer be maintained, with loss of MCA vasodilatation described in the most compromised fetuses (Figueroa‐Diesel et al., 2007). The posterior VA is less well studied in FGR fetuses, but abnormal VA flow appears confined to the most severely affected fetuses and tends to occur with concomitant widespread changes in other major cerebral arteries, indicating it may reflect late, high‐risk pathophysiology and sensitivity for severe FGR (Morales Rosello et al., 2012; Steller et al., 2022). Steller et al (2022) compared Doppler differences across four cerebral arteries in fetuses with FGR or SGA, and showed that vasodilatation of the ACA was the most common abnormal Doppler observation in the FGR group, while all fetuses with an abnormal VA Doppler had at least one other cerebral vessel abnormality and the highest incidence of perinatal morbidity.

Taken together, these observations have led to the hypothesis of a front‐to‐back pattern of cerebral artery vasodilatation that favours early frontal brain perfusion but progressively shifts with signs of fetal deterioration (Hernandez‐Andrade et al., 2008; Steller et al., 2022). Conceptually, this would underpin regional variability in response to utero‐placental dysfunction in FGR (Fig. 1). This brain blood flow distribution pattern is described in severe early‐onset FGR where umbilical artery abnormalities are prominent (Figueroa‐Diesel et al., 2007; Hernandez‐Andrade et al., 2008). A similar pattern is reported in late‐onset FGR, although umbilical artery abnormalities may be less marked (Morales Rosello et al., 2012; Steller et al., 2022). Observations supporting the front‐to‐back pattern of vasodilatation are shown in studies evaluating FGR fetuses in a longitudinal manner over time (Figueroa‐Diesel et al., 2007), or when FGR status is categorised by the severity of haemodynamic deterioration (Hernandez‐Andrade et al., 2008). Vasodilatation of the ACA aligns with prioritisation of oxygen supply to cortical and subcortical regions of the frontal lobe. With worsening compromise, vasodilatation of the PCA favours blood supply to the occipital and temporal lobes and deep grey matter including the thalamus and hippocampus. With an advancing state of fetal hypoxia, vasodilatation of the MCA favours lateral cortical blood supply of the parietal and temporal lobes and mid‐brain deep grey matter, which occurs concurrent with reduced perfusion to the frontal lobe (Figueroa‐Diesel et al., 2007; Hernandez‐Andrade et al., 2012). In severe fetal hypoxaemia, posterior circulation changes in the VA (Morales Rosello et al., 2012; Steller et al., 2022) may indicate sparing of brainstem perfusion. At this stage, observations of front‐to‐back cerebral vasodilatation with worsening FGR simply reflect the regional arterial responses to hypoxaemia. The physiological context and regional metabolic or cellular responses remain unknown (Morales Rosello et al., 2012); for example, given the critical nature of brainstem function, it is unlikely that key brainstem nuclei only receive extra oxygen supply under conditions of severe, terminal compromise, and therefore understanding how Doppler findings fit with regional physiological changes and/or neuropathology would benefit from further studies.

## The fetal haemodynamic response to hypoxia: acute mechanisms

### Peripheral response to acute hypoxia

The brain, and particularly the developing fetal brain, has the highest metabolic activity and energy expenditure of any organ in the body. Glucose is the brain's main fuel substrate and its efficient utilisation is critically oxygen‐dependent (Vannucci & Vannucci, 2000). Consequently, a fall in fetal oxygenation induces an immediate haemodynamic response to maintain oxygen delivery to this highly demanding organ, and while this is termed brain sparing, this is a multisystem response involving fetal cardiac output redistribution not only to the brain, but also to the heart and adrenals (Giussani, 2016). Preclinical studies in large animals, primarily sheep, greatly enhance our understanding of the fetal response to hypoxaemia, permitting unique monitoring access to real‐time changes in fetal physiology (Allison et al., 2026), which are foundational to our understanding of the immediate haemodynamic responses to acute hypoxia (Bennet et al., 1998; Bocking et al., 1988; Giussani et al., 1993). However, there is also growing recognition that acute hypoxia can manifest in various forms, with nuanced physiological responses observed depending on the type, timing, and severity of the hypoxic insult. Hypoxaemia is first detected by the fetal carotid body chemoreceptors, inducing a reflex afferent sinus nerve firing to signal hypoxaemia to the brainstem, and increasing sympathetic outflow to cause peripheral vasoconstriction and modulating heart rate, vascular tone and endocrine responses (Giussani et al., 1993; Kane et al., 2020). Notably, the carotid body is uniquely developed to detect hypoxaemia (arterial oxygen pressure, $P_{aO_{2}}$) and therefore exclusively initiates the set of actions that comprise the brain sparing response (Kane et al., 2020). Altered vascular tone and endocrine activation then represent secondary, more sustained responses to acute hypoxia. Sympathetic activation initially results in bradycardia and peripheral vasoconstriction, which centralises fetal cardiac output to the brain and increases mean arterial pressure (Giussani et al., 1993; Kane et al., 2020). In doing so, brain sparing ensures that in response to acute hypoxia, cerebral blood flow is maintained. However, brain perfusion is governed by hierarchical, locally regulated mechanisms rather than global cerebral vasodilatation, meaning that there is a temporal evolution of brain arterial response that selectively moderates cerebral perfusion in response to acute and chronic hypoxia (Hernandez‐Andrade et al., 2008).

### Cerebral blood flow response to acute hypoxia

Preclinical studies demonstrate the physiology of the acute brain sparing response to fetal hypoxaemia. Acute fetal hypoxaemia, from 10 to 60 min in late‐preterm to term‐equivalent age fetal sheep, rapidly increases cerebral perfusion, indicated by increased carotid blood flow and reduced carotid vascular resistance (Bennet et al., 1998; Blood et al., 2002; Giussani et al., 1993; Hunter, Blood, Power et al., 2003; Lee et al., 2009). The cerebral blood flow response is, however, dependent on the severity and type of hypoxic stressor, with acute severe, rapid fetal asphyxia induced by complete umbilical cord occlusion causing an immediate decrease in cerebral blood flow with increased resistance (Bennet et al., 1998; Hunter, Blood, Power et al., 2003). Even in the presence of brain sparing (elevated brain blood flow), cortical oxygen levels are still reduced, suggesting that circulatory redistribution mitigates but does not fully compensate for cerebral hypoxia. This was elegantly shown in a fetal sheep study by Hunter, Blood, Power et al. (2003) who used maternal inhalation of hypoxic gas (10% oxygen) while measuring fetal cortical tissue oxygenation with laser Doppler probes. Exposure to hypoxia elicited effective brain sparing in the fetus with increased cortical flow; however, cortical tissue $P_{O_{2}}$ fell from ∼8 Torr at baseline to ∼1 Torr during hypoxia, with cortical tissue oxygenation dropping further in the presence of complete cord occlusion (Hunter, Blood, White et al., 2003). Similarly, Lee et al. (2009) reported a rapid decline in cortical tissue oxygen partial pressure in fetal sheep exposed to isocapnic hypoxia (Lee et al., 2009). Thus, brain sparing preserves cerebral blood flow in response to moderate fetal hypoxaemia but does not ensure maintenance of tissue oxygenation. Severe and rapid fetal asphyxia exceed the limits of the fetus to maintain cerebral autoregulation, by inducing reduced return of flow to the heart, hypotension and reduced cerebral blood flow (Bennet et al., 1998; Bierwirth et al., 2025; Hunter, Blood, White et al., 2003).

### Mechanisms regulating cerebral vascular tone: local

While brain sparing redistributes fetal cardiac output to preferentially favour the cerebral circulation, the adequacy of local tissue oxygenation ultimately depends on dynamic local regulation of cerebral vascular resistance within the brain (Gagnon et al., 1997; Pearce, 2006). Vascular tone represents the net contractile state of a blood vessel, balancing constrictor and dilator influences arising from circulating factors, neural input, endothelial signals, intrinsic myogenic activity, and local metabolic conditions. Exposure of cerebral blood vessels to hypoxaemia elicits a rapid and regionally distinct change in the interstitial milieu that favours vasodilatation (Pearce, 2006). The nitric oxide (NO) pathway plays a central role in this vasodilatory response, and in normal pregnancy there is increased endogenous NO to maintain vasodilatory tone in the fetal brain (Miller et al., 1999). Vascular endothelial cells are key mediators, releasing NO as well as other factors such as prostanoids (Hunter, Blood, White et al., 2003). Hypoxic exposure raises endothelial Ca^2+^ via multiple pathways, leading to direct activation of endothelial nitric oxide synthase (eNOS), which is also upregulated by hypoxia‐inducible factor (HIF‐1α). Concurrently, hypoxia stimulates neuronal nitric oxide synthase (nNOS) expression in neurons (Van Mil et al., 2002). The resulting increase in NO production directly stimulates soluble guanylate cyclase, the enzyme that synthesises the second messenger cGMP, a potent vasodilator. Additional vasodilatory mediators contribute to the hypoxic response, including adenosine, vasopressin, pituitary adenylate cyclase‐activating peptide and endogenous opioids (Pearce, 2006). Notably, while α_1_‐adrenergic receptor activation regulates basal tone, it is not an essential mediator of hypoxia‐induced increase in cerebral blood flow, as pharmacological blockade with prazosin does not significantly alter the cortical perfusion response (Bierwirth et al., 2025). There is speculation about the role of vascular smooth muscle in the response to acute hypoxia; however, much of this work is inferred from adult literature and requires confirmation in the fetus. It is likely that fetal vascular smooth muscle has a reduced ability to generate the same tension as adult smooth muscle, and therefore provides less resistance against vasodilatation (reviewed in Pearce, 2006).

Although acute hypoxia is not always associated with elevated carbon dioxide (hypercapnia), hypoxia and hypercapnia frequently coexist. Hypercapnia elicits cerebral vasodilatation via local changes in extracellular pH around vascular smooth muscle cells (Yoon et al., 2012), thereby increasing cerebral blood flow by reducing cerebral vascular impedance. Additionally, hypercapnia has been shown to increase NO release from the cerebral vascular endothelium (Xu et al., 2004), enhancing local vasodilatation.

### Mechanisms regulating cerebral blood flow: metabolic and endocrine

An acute reduction in cerebral oxygen delivery alters both neuronal activity and cerebral metabolic rate (Blood et al., 2002; Hunter, Blood, Power et al., 2003; Lee et al., 2009). The magnitude of metabolic suppression varies with hypoxic severity. During mild (12 mmHg) fetal hypoxaemia, cerebral oxygen consumption can be maintained, though neuronal activity is reduced, as reflected by a shift in cerebral electrocorticography (ECoG) toward high‐voltage signals (Lee et al., 2009). Mild fetal hypoxia is also found to maintain a normal glucose‐to‐oxygen utilisation ratio, combined with an absence of lactate efflux in the sagittal sinus, which indicates preservation of predominantly oxidative metabolism, albeit at a reduced rate (Hunter, Blood, Power et al., 2003).

As the severity of acute fetal hypoxia increases, this compensatory capacity is exceeded, and cerebral metabolism is downregulated. In sheep exposed to maternal hypoxic gas inhalation (10–12% oxygen), fetal cortical oxygen consumption is reduced by 40%, while a rapid asphyxic insult (complete cord occlusion) results in a reduction approaching 90% (Hunter, Blood, Power et al., 2003). Critically, during a severe insult, the metabolic profile shifts; lactate is produced, and the glucose/oxygen quotient increases markedly, indicating a transition towards anaerobic glycolysis to maintain essential metabolic functions (Hunter, Blood, Power et al., 2003). Metabolic suppression is mediated, at least in part, by activation of adenosine A_1_ receptors (Blood et al., 2002), highlighting a key molecular mechanism underpinning the metabolic component of brain sparing. Altered cerebral metabolism during acute severe hypoxia can be detected using magnetic resonance spectroscopy (MRS), with an increase in the lactate: *N*‐acetylasparate (NAA) ratio serving as a biomarker of impaired oxidative metabolism (Aridas et al., 2021). Furthermore, elevated lactate: NAA ratio is shown to predict adverse neuropathological outcomes in the neonatal brain (Mitra et al., 2019), providing a valuable non‐invasive tool for assessing hypoxic injury severity and prognosis.

## The fetal haemodynamic response to hypoxia: chronic mechanisms

### Cerebral response to chronic hypoxia

**Figure 2:**
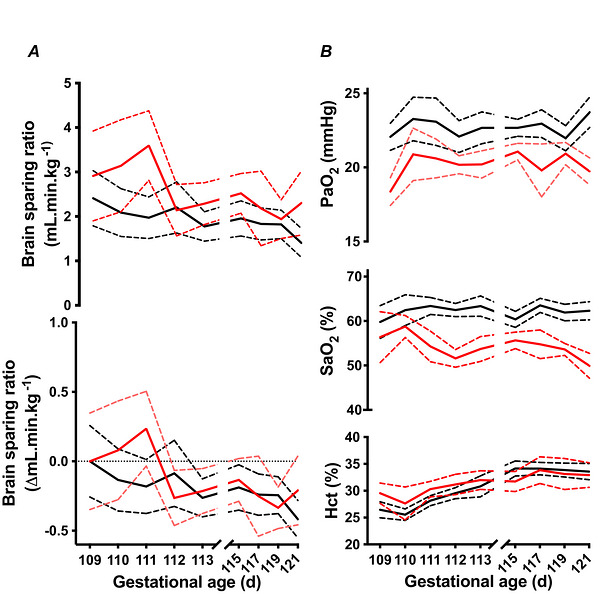
Brain sparing ratios and hypoxaemia status in ovine fetuses *A*, reanalysis from Inocencio et al. (2020) of brain sparing ratio (carotid‐to‐femoral blood flow, normalised to brain and fetal weight, respectively; top) and baseline‐corrected brain sparing ratio (normalised to 109 gestation age (days) values, ovine term 148 days; bottom), highlighting enhanced cerebral redistribution in fetal growth restriction (FGR, red) *versus* controls (black). *B*, fetal arterial partial pressure of oxygen ($P_{aO_{2}}$), saturation of oxygen ($S_{aO_{2}}$) and haematocrit (Hct) profiles from 109 to 121 days. FGR was induced by single umbilical artery ligation. Data illustrate progressive hypoxaemia in FGR and brain sparing as an adaptive response to hypoxaemia. Data shown are means ± SEM (dashed lines).

While the brain sparing response is best characterised under conditions of acute hypoxia (minutes), longer term preclinical studies have examined the physiological adaptations necessary to sustain brain sparing. Studies from the Ontario group in the late 1980s–1990s demonstrated a sustained increase in cerebral blood flow above control levels for up to 48 h during chronic moderate hypoxaemia (Bocking et al., 1988; Richardson, Carmichael et al., 1989; Richardson, Rurak et al., 1989; Rurak et al., 1990). Bocking et al. (1988) showed that 48 h of fetal hypoxaemia (40% reduced fetal O_2 s_aturation) increased cerebral blood flow across all regions of the brain examined, including the cortex, subcortex, pons, medulla and cerebellum. Interestingly, the increase in flow to the pons, medulla and subcortex was greater at 1 h after onset of hypoxaemia than at 24 and 48 h, which the authors hypothesised was influenced by an early elevation in arterial CO_2_, since the brainstem is more sensitive to changes in CO_2_ than other brain regions (Bocking et al., 1988). Fetal heart rate initially fell via vagal mechanisms, then rebounded by 10–15% for 12–16 h, possibly due to sustained catecholamine release (Bocking et al., 1988). Six hours of fetal hypoxaemia induced by placental embolisation caused an increase in cerebral blood flow and a decrease in cerebral vascular resistance (Gagnon et al., 1997), even in the face of worsening fetal metabolic acidosis. Towards the end of the 6‐h period there was a disconnect between reduced carotid blood flow (measured with flowprobes) compared to a sustained increase in total cerebral blood flow (measured with microspheres) that suggested vasodilatation of an alternate arterial source, such as the vertebral–basilar supply; this is supported by their observation that spinal cord and brainstem regional flow showed the greatest sustained elevation (Gagnon et al., 1997). An 8 h total period of fetal hypoxaemia induced via maternal hypoxia increased cerebral blood flow at 1–2 h to a similar degree as observed with acute hypoxaemia, although this was followed by a terminal fall in cerebral flow at 8 h that may have been due to decreased arterial pressure (Rurak et al., 1990). Over a longer period of chronic fetal hypoxaemia induced by single umbilical artery‐induced placental insufficiency and FGR, with fetal oxygenation reduced by ∼16% and elevated haematocrit, total brain blood flow was halved (Miller et al., 2009). This was not, however, uniform, with profound regional blood flow changes in cerebral grey and white matter, but relative preservation of brainstem perfusion (Miller et al., 2009). In the same model (Inocencio et al., 2020), we have subsequently reanalysed longitudinal data and found an initial increase in carotid artery blood flow, followed by normalisation towards control levels over 16 days after the onset of placental insufficiency (Fig. 2). More recently, Darby et al. (2024), using a well‐established sheep model of early‐onset placental insufficiency to induce FGR with moderate fetal hypoxaemia (∼20% reduction), applied phase‐contrast MRI plus T2 oximetry to quantify fetal blood flows during a superimposed acute hypoxaemia ‘second hit’. In chronically hypoxaemic FGR fetuses, Darby et al. (2024) reported that carotid artery flow was not different in FGR and control fetuses, consistent with our longitudinal findings in Fig. 2, and nor was cerebral oxygen delivery or consumption, which implies that other factors such as hormonal or metabolic substrates may provide compensation. Taken together, these studies support that an initial cerebrovascular dilatation occurs in mild–moderate fetal hypoxaemia; however, this is not sustained over days to weeks, at least in the anterior cerebral supply, but the sustained presence of brain sparing is supported by prolonged elevation of regional brain blood flow to specific brain regions including the brainstem. Furthermore, as chronic hypoxia continues or worsens, fetal adaptations extend beyond cerebral vasodilatation alone and increasingly rely on broader redistribution of the circulation to preserve oxygen delivery to vital organs. Darby et al. (2024) identified increased ductus venosus (DV) flow with greater right‐to‐left shunting via the foramen ovale (FO) in FGR fetal sheep, consistent with enhanced DV–FO streaming of oxygenated umbilical venous blood toward the left heart and upstream to the cerebral circulation. However, even with this increased DV–FO pathway maintained during the acute hypoxaemic ‘second hit’, cerebral oxygen delivery fell, indicating that preferential streaming can bolster oxygen distribution under chronic compromise but may be insufficient when an additional acute insult is superimposed (Darby et al., 2024).

**Figure 3:**
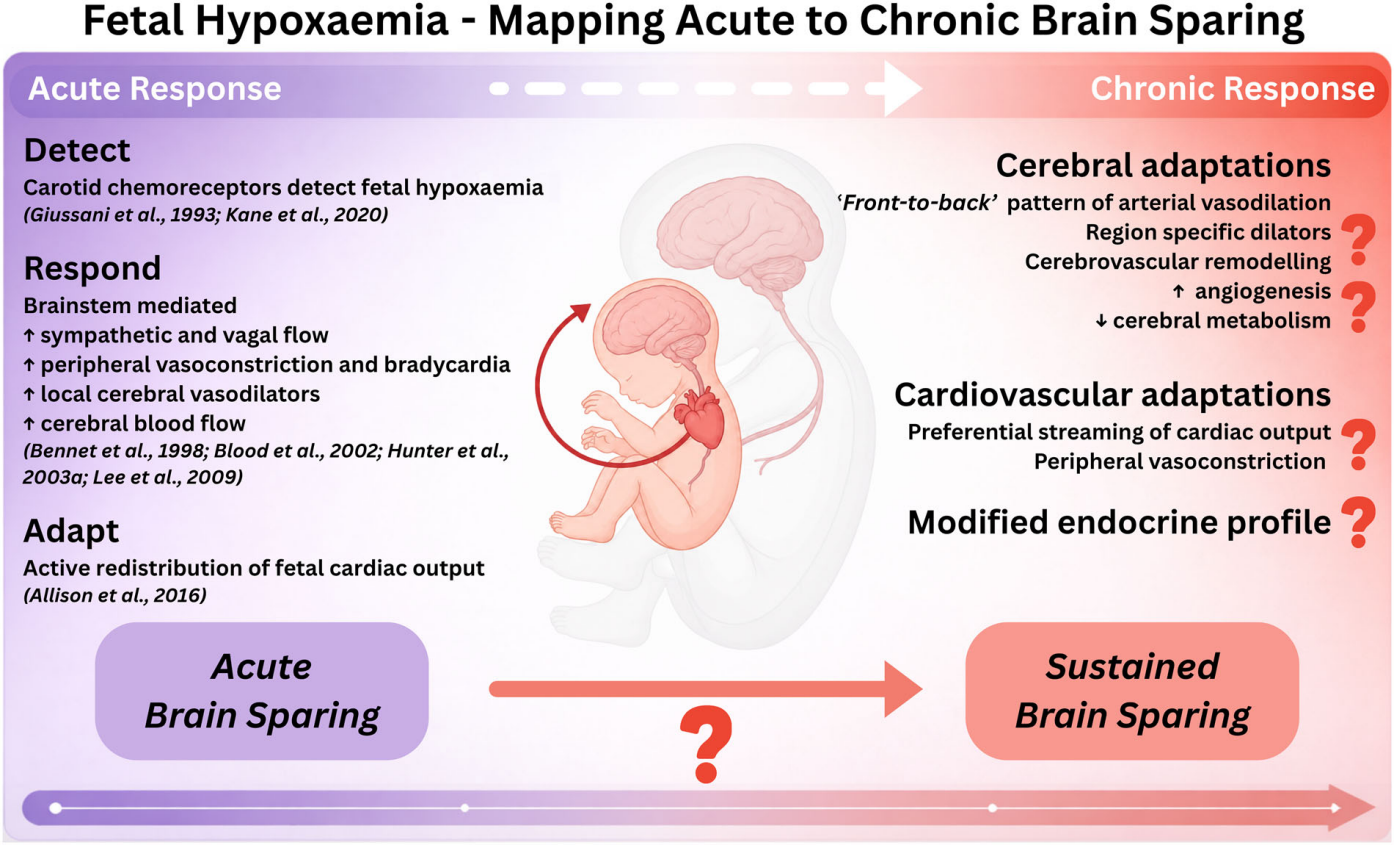
Fetal hypoxaemia—mapping acute to chronic brain sparing The left panel provides an overview of the acute physiological responses to fetal hypoxaemia, incorporating the detection, active response and adaptation. In FGR, the sustained brain sparing response to chronic fetal hypoxaemia is less well characterised (right panel), with proposed adaptations spanning cerebral, cardiovascular, and modified endocrine profile.

Additionally, in fetal sheep exposed to maternal isobaric hypoxia (10%, from 121 to 132 days of gestation) in free‐roaming, non‐stressed ewes (conditions maintained through continuous wireless data acquisition), oxygen delivery to the fetal brain was progressively prioritised over the periphery (Allison et al., 2016). In sheep pregnancies maintained at high altitude for 100 days (from 30 to 130 days’ gestation), fetal heart right ventricular function was severely impaired, reducing right ventricular output and cardiac output (Kamitomo et al., 1992). In studies using a similar model, a single time point assessment of cardiac output showed preserved flow to the brain and heart, and reduced flow to the kidney, gastrointestinal tract and limbs (Kamitomo et al., 1993). These findings demonstrate that chronic hypoxia involves not only cerebral adaptation, but also redistribution away from peripheral organs to preserve oxygen and substrate delivery to vital tissues. Chronic fetal hypoxia also induces cerebrovascular remodelling that promotes angiogenesis (new blood vessel formation) (Pearce, 2006), but with compromised blood vessel structure arising from suboptimal development of the cells that comprise the neurovascular unit (Wixey et al., 2026). In high altitude sheep pregnancies, fetal cerebral arteries exhibit diminished capacity to generate force (e.g., vasoconstriction) through both vascular smooth muscle and endothelium‐derived mechanisms (Longo et al., 1993), which may impair cerebrovascular autoregulation. This structural remodelling likely reflects an adaptation to sustained vasodilatation but also represents maladaptive changes that increase perinatal susceptibility to brain injury. As outlined in Fig. 3, while the acute mediators of the brain sparing response are well defined, the persistent adaptations to prolonged fetal hypoxaemia in FGR are less well characterised.

In a mouse model of chronic maternal hypoxia, fetal cerebral perfusion was maintained through decreased cerebrovascular impedance, via preferential streaming of umbilical venous blood across the DV and through the FO towards the cerebral circulation (Cahill et al., 2019). Although the mechanisms contributing to sustained cerebrovasodilatation remain unclear, Jensen et al. (2005) reported increased expression of adrenomedullin, a vasodilator peptide, which they postulate may contribute to cerebral vasodilatation. The longer‐term contributions of key vasodilators identified in acute studies, including the NO pathway, are of interest, but results from Paz et al. (2019) indicate that FGR is associated with impaired carotid artery reactivity to NO in adult guinea pigs. Overall, these studies support the concept that brain sparing during chronic hypoxia is maintained by multiple coordinated circulatory and systemic adaptations, including cerebrovascular dilatation, preferential streaming of cardiac output, redistribution away from peripheral organs, metabolic and endocrine mechanisms. In response to prolonged or worsening fetal hypoxaemia, these mechanisms appear to evolve from adaptive to maladaptive; however, the dynamic changes that characterise this protective to pathological state require elucidation (Fig. 3).

### Metabolic and endocrine control of the chronic response to hypoxia

Beyond haemodynamic redistribution, chronic hypoxia induces systemic compensatory mechanisms to enhance blood oxygen‐carrying capacity, including increased fetal haemoglobin, haematocrit, fetal blood volume, erythrocyte volume and plasma erythropoietin concentration (Allison et al., 2016; Kitanaka et al., 1989).

In the brain, glucose catabolism, oxygen consumption and cerebral perfusion are tightly coupled. In the adult brain, when oxygen delivery is reduced, ATP is converted to ADP, AMP and then adenosine, the latter of which is released from all cell types of the brain (Kuschinsky, 1991). Adenosine binds to adenosine A2a receptors in the brain, promoting vasodilatation. A study in fetal sheep at 80% gestation demonstrated the importance of adenosine in the acute hypoxic response. Giussani and colleagues exposed fetuses to hypoxaemia with and without adenosine antagonism (Giussani et al., 2001), but the role of this nucleoside in chronic exposure to hypoxaemia *in utero* remains unexplored. Chronic fetal hypoxia reportedly induces a sustained global reduction in oxygen consumption (per kilogram body weight) and a proportional decrease in energy utilisation (Cetin et al., 2020; Mando et al., 2014). This metabolic suppression is associated with reduced tissue growth rate (and thus growth restriction) and diminished fetal activity, representing an adaptive strategy to match metabolic demand to reduced oxygen availability (Richardson & Bocking, 1998). Cerebral metabolism and brain activity are tightly coupled, with changes in neuronal activity mediating the brain's energy demands. FGR is associated with decreased brain activity, as seen with a reduction in rapid eye movement (REM) sleep and fetal breathing movements (Pulgar et al., 2007). REM sleep state is associated with relatively high cerebral metabolic demand, and therefore spending less time in this state reduces metabolic expenditure and conserves oxygen under conditions of limited supply (Lenzi et al., 1999).

The hypothalamic–pituitary–adrenal (HPA) axis is critical for regulating glucocorticoids, which, in turn, drive the differentiation and maturation of multiple organs during gestation. Under conditions of chronic hypoxia, persistent activation of the HPA axis enhances cortisol release, a response that contributes to systemic effects such as hyperglycaemia, bone loss and hypertension. However, despite this activation, the fetal cortisol response to adrenocorticotropic hormone stimulation is blunted, potentially compromising stress responsiveness (Kitanaka et al., 1989). The specific circulating changes in hormonal regulation are highly dependent on the type, timing and severity of chronic insult and have been summarised in a review by Newby et al. (2015). Within the developing brain, these hormonal alterations coincide with increased expression of atrial and brain natriuretic peptides, reflecting neuroendocrine compensation to hypoxic stress. The metabolic response of the fetal brain to hypoxia has also been well documented in the fetal physiology literature, showing a decrease in metabolic rate (Jones et al., 1983; Llanos et al., 2007). Consistent with this adaptation, genes related to energy metabolism, including those governing oxidative phosphorylation and glucose metabolism, are downregulated in the fetal hypothalamus (Wood et al., 2013), indicating a shift toward energy conservation during prolonged oxygen deprivation. As mentioned earlier with respect to acute perinatal asphyxia, there is a reduction in brain NAA on MRS imaging (Aridas et al., 2021), and while less well studied, MRS of the fetal brain of human FGR infants shows reduced NAA: creatine and NAA: choline ratios, likely attributable to reduced neuronal viability or neuronal mitochondrial dysfunction (Story et al., 2011). This is supported by a preclinical rabbit model of FGR in which a targeted metabolomics approach showed that cerebral NAA is reduced, alongside disruption to neurotransmitter peptides, amino acids, fatty acids, energy metabolism intermediates and oxidative stress‐related metabolites (van Vliet et al., 2013). In turn, cellular‐level metabolic dysfunction is likely to underpin poor neurodevelopment with reduced dendritogenesis and synaptic signalling (Dudink et al., 2025; White et al., 2026), accompanied by increased reactive oxygen species production and shifting oxidative balance towards oxidative stress (Malhotra et al., 2024; Miller et al., 2007, 2014). These results bring together evidence of a coordinated prolonged response in the FGR fetus that does not simply reflect elevated global cerebral blood flow, but rather shows cerebrovascular remodelling and region‐specific perfusion changes, alongside significant changes to brain metabolism and neuroendocrine regulation.

## Brain sparing is associated with adverse perinatal and neurodevelopmental outcomes

Both the terminology and concept of brain sparing imply a protective benefit to the brain. However, in human pregnancy, the presence of brain sparing is used as a diagnostic marker of FGR, and the severity of brain sparing can be linked to adverse perinatal outcomes and neurodevelopmental abnormalities. In human pregnancy, FGR with brain sparing is associated with perinatal death or perinatal morbidities that include preterm delivery, lower birthweight, neonatal acidosis, low APGAR score and more frequent admission to a neonatal intensive care unit (Coenen et al., 2022; Conde‐Agudelo et al., 2018; Cruz‐Martinez et al., 2011; Dubiel et al., 2002; Flood et al., 2014; Steller et al., 2022). FGR fetuses with brain sparing (elevated brain: liver volume ratio) demonstrate a 3.3‐fold increase in perinatal mortality risk (Damodaram et al., 2012). The same study showed that smaller lung volume in FGR fetuses predicted the need for continuous positive airway pressure (CPAP) postnatally, reflecting the need for greater respiratory support (Damodaram et al., 2012). Dubiel et al. (2002) examined blood flow velocity in the MCA, ACA and PCA in fetuses from hypertensive pregnancies and showed that abnormal ACA velocimetry was predictive for perinatal mortality. Consistent with the observation that abnormal MCA Doppler reflects worsening fetal condition, FGR fetuses with MCA vasodilatation are considered high risk in the perinatal period, with a reduced tolerance to intrapartum hypoxia and increased incidence of fetal distress (Cruz‐Martinez et al., 2011).

In a secondary analysis of the large TRUFFLE trial comparing antenatal monitoring strategies in early FGR, Stampalija et al. (2017) demonstrated that fetal MCA‐PI at study inclusion, but not shortly before birth, could predict 2‐year infant survival and neurodevelopment, but the authors note that gestational age at birth and birthweight were more powerful factors for determining neonatal and infant outcome (Stampalija et al., 2017). The TRUFFLE trial showed that despite some associations with adverse outcome, none of the brain sparing measures of MCA‐PI, CPR or UCR allowed predictive targeting for the optimal time of delivery in early onset FGR (Stampalija et al., 2017). A number of studies have examined whether SGA, FGR and/or FGR with brain sparing increases the risk of intraventricular haemorrhage (IVH) in the newborn period in preterm infants (Baschat et al., 2002; Flood et al., 2014; Meher et al., 2015; Piscopo et al., 2025); most studies report that brain sparing is not a risk factor for IVH; however, discrepancies in gestational age at birth make comparisons difficult given the strong association between earlier gestation at birth and IVH, with one study showing that periventricular echodensities indicative of lesions were increased in infants with antenatal cerebral redistribution (Habek et al., 2004).

The presence of brain sparing in FGR fetuses is strongly associated with adverse changes to brain structure and function. Antenatally, FGR fetuses display a reduced degree of brain gyrification and brain volume compared to appropriately grown fetuses, and FGR fetuses with brain sparing show reduced gyrification compared to FGR infants without brain sparing (Yehuda et al., 2024). FGR fetuses with brain sparing have significantly reduced total brain volume compared to appropriately grown controls (Maunu et al., 2007; Meijerink et al., 2025). Two systematic reviews describe the impact of brain sparing and cerebral vasodilatation on neurodevelopmental outcome; both Meher et al. (2015) and Benitez‐Marin et al. (2021) broadly agree that brain sparing does not protect the brain from injury, but both also note a heterogeneity of studies related to the inclusion criteria for FGR, infant age at neurodevelopmental assessment, and assessment outcomes. The presence of MCA redistribution in late pregnancy is associated with abnormal neurobehaviour scores, including motor function, that are detectable from term‐equivalent age (Oros et al., 2010). When utero‐placental dysfunction (abnormal UA impedance) is observed in pregnancy, 50–60% of fetuses will develop neurological deficits in childhood (Fouron et al., 2005). This concurs with the findings of Eixarch et al. (2008) who found that neurodevelopmental impairments were observed at 2 years of age in 52% of children who had MCA‐PI <5% percentile during fetal life, predominantly attributed to poor communication and problem solving in the infants with brain sparing (Eixarch et al., 2008). Monteith et al. (2019) compared childhood neurodevelopment at 3 years of age across multiple cohorts who were SGA at birth, FGR with abnormal UA Doppler but normal CPR, or FGR with abnormal UA Dopplers and abnormal CPR (i.e., with brain sparing), and found that brain sparing significantly increased the risk for poor motor, language and cognitive outcomes (FGR with abnormal UA *vs*. FGR with abnormal UA and abnormal CPR).

The ACA principally supplies the cortical and subcortical regions of the frontal lobe, a brain region that is integral to cognition. With increasing fetal deterioration, there occurs a shift from preferential perfusion of the ACA to the MCA, thereby leaving the frontal brain vulnerable. A reduction in perfusion to frontal regions would likely result in reduced grey matter cortical development, and this has indeed been observed in MRI measures of brain volume in FGR infants and children (Dubois et al., 2008; Tolsa et al., 2004). In turn, these structural deficits in the frontal cortex would be expected to underpin reduced cognitive capacity in children who were born FGR with brain sparing (Geva et al., 2006; Morsing et al., 2011). Geva et al. (2006) analysed birth characteristics with neuropsychological evaluation at 9 years of age, and showed that FGR with brain sparing (head circumference) was associated with reduced intelligence quotient (IQ), and deficits in executive function, flexibility–creativity and language that were indicative of compromised frontal lobe development. While abnormal MCA flow detects a more advanced haemodynamic state in the FGR brain, ACA vasodilatation, and therefore preferential blood flow to the frontal region, is also associated with deficits in neurodevelopmental behaviour soon after birth, and increased incidence of emotional and attention problems at 18 months of age (Cruz‐Martinez et al., 2009; Roza et al., 2008). Finally, Scherjon et al. have followed and assessed a cohort of early‐onset FGR infants with brain sparing across multiple childhood time points, and showed that at 3 years of age brain sparing was not linked to neurodevelopmental outcome (Scherjon et al., 1998), but at 5 years of age fetal brain sparing predicted a reduced IQ score (Scherjon et al., 2000) and at 11 years of age >20% of FGR‐born children had behavioural problems, but this was more strongly related to prematurity than brain sparing (van den Broek et al., 2010). This raises the important point that particularly in early‐onset FGR, even while severely abnormal utero‐placental Dopplers independently contribute to poor neurodevelopmental outcomes, gestational age and birth weight remain primary mediators of altered brain structure and function (Baschat et al., 2009).

These results indicate that the term brain sparing does not adequately describe the dynamic nature of the acute to chronic response and that severe brain sparing, with prolonged MCA vasodilatation or VA vasodilatation, is associated with neuropathology. FGR human fetuses with MCA vasodilatation have significantly elevated risks for perinatal death or neonatal morbidities, with a greater degree of brain sparing associated with poorer outcome. Thus, brain sparing should primarily be considered a marker of fetal wellbeing associated with utero‐placental dysfunction. Brain sparing does not guarantee normal brain development. While redistribution may transiently support oxygen delivery to critical brain regions, a sustained response mediates a hierarchical redistribution of cerebral blood flow, and significant changes in cerebral metabolism, vascular development, neurotransmitter release and oxidative balance. Each of these factors is likely to contribute to increased susceptibility to neuropathology, although it is not known whether modifying any or all could improve brain development and function.

## Brain sparing is associated with altered cardiovascular and metabolic programming

The consequences of the brain sparing response in the FGR fetus extend beyond the brain, with the same adaptations that support short‐term survival associated with altered cardiovascular and metabolic health. As reviewed by Crispi et al. (2018), cardiovascular remodelling associated with FGR determines cardiovascular function and dysfunction for life. Studies in this area were first described in the late 1980s with the landmark studies of David Barker demonstrating that low birth weight was inversely related to systolic blood pressure in children and adults (Barker, Osmond, Golding et al., 1989), and elevated risk of stroke or ischaemic heart disease (Barker, Osmond, Winter et al., 1989). For these studies, Barker employed low birth weight as a proxy for suboptimal intrauterine growth and proposed ‘A relationship between intrauterine influences and cardiovascular disease’ (Barker, Osmond, Golding et al., 1989), today called the *Developmental Origins of Health and Disease* (DOHaD). Epidemiological evidence from Sweden found a significant association between low birth weight and death from ischaemic heart disease in men over 65, with adjustment for socioeconomic status (Leon et al., 1998). Similarly, a cohort study from Sheffield, UK demonstrated that for every ∼450 g decrease in birth weight, systolic blood pressure in adulthood increased by 2.7 mmHg (Martyn et al., 1995). This relationship remained robust after accounting for sex, body mass index, alcohol intake and gestational age (Martyn et al., 1995). In an Australian context, Indigenous Australians born between 1992 and 1998 in the lowest birthweight quartile exhibited twice the risk of cardiovascular disease in adulthood compared to those in the highest quartiles (Arnold et al., 2015). The strong association between hypoxia during pregnancy, FGR and adverse cardiovascular outcomes has been well reviewed (Crispi et al., 2018; Giussani, 2021; Rock et al., 2021), with good evidence from animal studies showing that heart and vascular dysfunction is detectable in FGR offspring as early as the neonatal period, and in adolescence, preceding the onset of adult cardiovascular disease (Azman et al., 2025; Botting et al., 2018; Brain et al., 2019; Patey et al., 2025; Rock et al., 2023).

Preclinical studies provide important mechanistic insight to validate the epidemiological data. Chronic exposure of pregnant ewes to hypoxia in an isobaric chamber drove a shift from the expected late gestation right ventricular dominance to a marked left ventricular dominance and global left ventricular dysfunction in the developing fetal heart (Patey et al., 2025). These functional changes were associated with cardiac structural changes that included left ventricular sphericity and biventricular hypertrophy (Patey et al., 2025). Patey and colleagues found that with increasing duration of maternal hypoxic exposure, the fetal left ventricle initially augmented contractile deformation, but this adaptive response was followed by the emergence of right ventricular dysfunction, indicating that fetal myocardial compensation to sustained hypoxia is finite and ultimately becomes exhausted (Patey et al., 2025). In FGR newborns, distinct alterations have been identified in cardiac structure, such as thickened interventricular septum and ventricular free walls, resulting in a more globular, hypertrophied heart shape (Rock et al., 2023; Rock et al., 2026; Sehgal et al., 2017). These structural abnormalities are closely linked to impaired cardiac function, with meta‐analyses showing significant systolic and diastolic dysfunction, particularly of the left ventricle (van de Meent et al., 2024). Furthermore, maladaptive ventricular‐arterial coupling has been observed in FGR newborns, where increased vascular stiffness and resistance are significantly correlated with cardiac dysfunction (Sehgal et al., 2018). Crispi et al. (2012) compared cardiovascular morphology in 3‐ to 6‐year‐olds who were categorised as control, SGA (no brain sparing) or FGR (brain sparing) and showed that SGA and FGR children were similarly likely to have an altered heart shape, increased stroke volume, cardiac output, left ventricular thickening and elevated blood pressure compared to control children. Beyond the cardiovascular system, FGR also increases the risk of ongoing metabolic dysregulation in the postnatal period. FGR offspring tend to have lower lean body mass but increased adiposity (Hesse et al., 2022), as well as increased hepatic insulin resistance (Rozance & Hay, 2010) and an overall increased risk of ongoing metabolic disturbances (Gilley et al., 2024). Accordingly, it is important to recognise that brain sparing is not simply an adaptation that has long term effects on the brain, but there is also certainly room to further elucidate how prolonged brain sparing mediates cardiac, vascular and metabolic programming.

There are limited data on how cardiovascular outcomes vary according to the degree of brain sparing in FGR. Existing studies suggest that cardiac function worsens as brain sparing becomes more pronounced; prospective cohort studies have demonstrated that myocardial performance index (Kaya et al., 2026) and cardiac timing intervals, such as ejection timing and mitral *E*/*A* velocities (Arslanoglu et al., 2025), worsen as the CPR decreases. One of the most comprehensive studies followed 200 children identified with FGR in fetal life until childhood, with FGR determined via umbilical artery Doppler assessment and categorised as mild (PI <2 SD) or severe (PI >2 SD) (Crispi et al., 2010). Children with FGR demonstrated marked cardiac and vascular remodelling, including significantly reduced stroke volume and impaired diastolic relaxation, with the most pronounced changes observed in those with severe FGR (Crispi et al., 2010). Collectively, these findings indicate a potential relationship between the degree of brain sparing and subsequent cardiovascular remodelling, although CPR‐stratified outcome data remain scarce. Physiologically, increased placental and systemic vascular resistance would be expected to increase right ventricular afterload, whereas conversely, the left ventricle would be exposed to increased preload and relatively reduced afterload to the cerebral circulation. The magnitude of these altered loading conditions on the fetal heart is likely to contribute to measurable changes in perinatal and long‐term cardiac function.

## Summary and future directions

The fetus is exquisitely sensitive to low blood oxygen, detecting hypoxaemia and mounting a rapid physiological response to preferentially shunt cardiac output to the brain, adrenals and heart. This brain sparing response requires a series of coordinated events initiated by the fetal carotid chemoreceptors to sense hypoxaemia and signal a brainstem‐mediated increase in sympathetic and vagal outflow to cause peripheral vasoconstriction and bradycardia, while local release of vasodilator substrates mediates increased cerebral blood flow (Fig. 3) (Giussani, 2016; Giussani & Davidge, 2013). Importantly, the carotid body is uniquely sensitive to low arterial oxygen pressure ($P_{aO_{2}}$) and therefore the brain sparing response is a specific reaction to hypoxaemia (Kane et al., 2020). In the short‐term, the brain sparing response preserves cerebral oxygen delivery.

In normal fetal brain development, the major cerebral arteries naturally expand across late gestation to accommodate increasing cerebral blood flow (Kulenovic & Dilberovic, 2004). In cases of prolonged or worsening fetal hypoxaemia, the brain sparing response evolves into a dynamic blood flow redistribution (Hernandez‐Andrade et al., 2008). The brain sparing response appears to begin with preferential vasodilatation of the anterior cerebral territories to protect cortical and subcortical flow, and progresses to include middle, posterior and vertebro‐basilar artery vasodilatation as fetal hypoxia becomes prolonged or severe. This apparent front‐to‐back pattern of cerebral vasodilatation initially protects higher cognitive functions, followed by a shift towards preservation of basic survival with prioritisation of brainstem flow during severe deterioration. The mechanisms that maintain chronic fetal brain sparing require full elucidation but likely involve a series of longer‐term events that include cerebrovascular and cardiovascular remodelling, preferential streaming of cardiac output, sustained peripheral vasoconstriction, and a significantly modified metabolic and endocrine environment.

Experimental studies characterising the spectrum of dynamic changes in brain blood flow distribution during chronic fetal hypoxaemia would advance our understanding of the ‘double‐edged sword’ nature of prolonged brain sparing. Future directions should include longitudinal large‑animal studies in chronically instrumented fetal sheep, using carefully titrated and sustained hypoxaemia, to assess in real time how brain sparing evolves from acute redistribution towards a chronic adverse state, integrating continuous haemodynamic monitoring with measures of cerebral and peripheral perfusion. These models can be designed to mimic human placental insufficiency and are precisely the type of high‑rigour large‑animal research that has underpinned past advances but is now at risk, as highlighted by recent calls to protect the perinatal translational pipeline (Allison et al., 2026). Integrative studies that pair *in vivo* Doppler or functional MRI readouts with *ex vivo* molecular profiling of cerebral arteries would also directly link Doppler outcomes to underlying endothelial and perivascular changes. Finally, interventional paradigms that manipulate identified mediators of function (as undertaken in acute studies, e.g. Giussani et al., 1993) during chronic hypoxaemia would test whether targeting these pathways shifts the threshold or trajectory of the adaptive‐to‐maladaptive transition. Together, these experiments would move the field from describing brain sparing as simply present or absent, towards an understanding of the full spectrum of brain sparing and potentially enabling better detection and clinical management of growth restricted fetuses.

Importantly, although brain sparing is adaptive and protective in the short term, it does not necessarily confer cerebral protection. In human FGR, vasodilatation of the MCA (reduced MCA‐PI) is most often reported as the measure of brain sparing, but MCA vasodilatation probably reflects an advanced state of fetal hypoxaemia (Figueroa‐Diesel et al., 2007). In human infants with FGR, brain sparing is linked to adverse perinatal outcomes, altered brain development (Coenen et al., 2022; Conde‐Agudelo et al., 2018; Cruz‐Martinez et al., 2009; Dubiel et al., 2002; Flood et al., 2014), and poorer longer‐term neurodevelopmental performance, particularly in cognition and learning domains linked to cortical function (Stampalija et al., 2017). Increasing evidence also suggests that the consequences extend beyond the brain, with altered cardiovascular structure and function in the neonatal period and persisting into later life (Barker & Osmond, 1988; Crispi et al., 2018). Thus, brain sparing is best understood not simply as a protective phenomenon, but as a dynamic marker of fetal hypoxic compromise that shifts from adaptive to maladaptive with increasing duration and severity of hypoxaemia. The mechanisms, physiology and ontogeny of sustained brain sparing in severe FGR require further characterisation, but understanding the dynamic changes that mediate a protective to pathological state afford new opportunities to diagnose and manage FGR, and potentially also to intervene to prevent poor short‐ and long‐term consequences of FGR.

## Additional information

### Competing interests

The authors declare that they have no competing interests.

### Author contributions

All authors – B.J.A., T.A.W., E.J.C., and S.L.M. – worked together to conceive this work and contribute to all aspects of drafting and writing and all contributed intellectual content. All authors have read and approved the final version of this manuscript and agree to be accountable for all aspects of the work in ensuring that questions related to the accuracy or integrity of any part of the work are appropriately investigated and resolved. All persons designated as authors qualify for authorship, and all those who qualify for authorship are listed.

### Funding

Authors wish to acknowledge the funding bodies that have supported personnel and their work; these include National Health and Medical Research Council (NHMRC) Australia Investigator support to B.J.A. and S.L.M. (#1175843 and #2016688), the Heart Foundation (110612‐2025), and Inner Wheel Australia.

### Generative AI statement

Generative artificial intelligence (Notebook) was used in the creation of Figure 1 and the graphical abstract.

## Supporting information


Peer Review History
