# Supplementary material for: Brain sparing in fetal growth restriction: The double‐edged sword of fetal hypoxaemia
Source: J Physiol. 2026 Jul 6;604(15):6259–80. doi: 10.1113/JP291253 (PMC13423135; doi:10.1113/JP291253)
Supplement: Supplementary file 1 — Peer Review History [file TJP-604-6259-s001.pdf]

The Journal of Physiology

BRAIN SPARING IN FETAL GROWTH RESTRICTION: THE DOUBLE-EDGED SWORD OF FETAL HYPOXAEMIA

Beth J Allison, Tegan A White, Emily J Camm, and Suzanne L Miller
DOI: 10.1113/JP291253

Corresponding author(s): Suzanne Miller (suzie.miller@monash.edu)

The following individual(s) involved in review of this submission have agreed to reveal their identity: Dino A Giussani (Referee #1)

Review Timeline:

Submission Date:	23-Apr-2026
Editorial Decision:	27-May-2026
Revision Received:	04-Jun-2026
Accepted:	08-Jun-2026

Senior Editor: Laura Bennet

Reviewing Editor: Christopher Lear

Transaction Report:

(Note: With the exception of the correction of typographical or spelling errors that could be a source of ambiguity, letters and reports are not edited. Depending on transfer agreements, referee reports obtained elsewhere may or may not be included in this compilation. Referee reports are anonymous unless the Referee chooses to sign their reports.)

Dear Professor Miller,

Re: JP-TR-2026-291253 "**THE CAUSES AND CONSEQUENCES OF THE BRAIN SPARING RESPONSE IN FETAL GROWTH RESTRICTION**" by Beth J Allison, Tegan A White, Emily J Camm, and Suzanne L Miller

Thank you for submitting your manuscript to The Journal of Physiology. It has been assessed by a Reviewing Editor and by 2 expert referees and we are pleased to tell you that it is acceptable for publication following satisfactory revision.

The referee reports are copied at the end of this email.

Please address all the points raised and incorporate all requested revisions or explain in your Response to Referees why a change has not been made. We hope you will find the comments helpful and that you will be able to return your revised manuscript within 4 weeks. If you require longer than this, please contact journal staff: jp@physoc.org.

Your revised manuscript should be submitted online using the link in your Author Tasks: Link Not Available. This link is accessible via your account as Corresponding Author; it is not available to your co-authors. If this presents a problem, please contact journal staff (jp@physoc.org). Image files from the previous version are retained on the system. Please ensure you replace or remove any files that are being revised.

If you do not wish to submit a revised version of your manuscript, you must inform our journal staff (jp@physoc.org) or reply to this email to request withdrawal. Please note that a manuscript must be formally withdrawn from the peer review process at one journal before it may be submitted to another journal.

TRANSPARENT PEER REVIEW POLICY: To improve the transparency of its peer review process, The Journal of Physiology publishes online as supporting information the peer review history of all articles accepted for publication. Readers will have access to decision letters, including Editors' comments and referee reports, for each version of the manuscript, as well as any author responses to peer review comments. Referees can decide whether or not they wish to be named on the peer review history document.

ABSTRACT FIGURES: Authors may use The Journal's premium BioRender account to create/redraw their Abstract Figures (and any other suitable schematic figure). Information on how to access this account is here: https://physoc.onlinelibrary.wiley.com/journal/14697793/biorender-access.

This will enable Authors to create and download high-resolution figures. If authors have used the free BioRender service, they can use the instructions provided in the link above to download a high-resolution version suitable for publication. The link provided should only be used for the purposes of this submission. Authors will be charged for figures created on this account if they are not related to this manuscript submission.

LANGUAGE EDITING AND SUPPORT FOR PUBLICATION: If you would like help with English language editing, or other article preparation support, Wiley Editing Services offers expert help, including English Language Editing, as well as translation, manuscript formatting, and figure formatting at www.wileyauthors.com/eeo/preparation. You can also find resources for Preparing Your Article for general guidance about writing and preparing your manuscript at www.wileyauthors.com/eeo/prepresources.

REVISION CHECKLIST: Upload a full Response to Referees file. To create your 'Response to Referees' copy all the reports, including any comments from the Senior and Reviewing Editors, into a Microsoft Word, or similar, file and respond to each point, using font or background colour to distinguish comments and responses and upload as the required file type.

Please upload two versions of your manuscript text: one with all relevant changes highlighted and one clean version with no changes tracked. The manuscript file should include all tables and figure legends, but each figure/graph should be uploaded as separate, high-resolution files.

You may also upload:

- 'Potential Cover Art' for consideration as the issue's cover image
- Appropriate Supporting Information (Video, audio or data set: see https://jp.msubmit.net/cgi-bin/main.plex?form_type=display_requirements#supp).

We look forward to receiving your revised submission.

If you have any queries, please reply to this email and we will be pleased to advise.

Yours sincerely,

Laura Bennet
Senior Editor

REQUIRED ITEMS

- Please include an Abstract Figure file and an Abstract Figure legend. An appropriate figure legend, which should not exceed 150 words in length, should be included in the main manuscript file. The Abstract Figure is a piece of artwork designed to give readers an immediate understanding of the research and should summarise the main conclusions. If possible, the image should be easily 'readable' from left to right or top to bottom. It should show the physiological relevance of the manuscript so readers can assess the importance and content of its findings. Abstract Figures should not merely recapitulate other figures in the manuscript. Please try to keep the diagram as simple as possible and without superfluous information that may distract from the main conclusion(s). Abstract Figures must be provided by authors no later than the revised manuscript stage and should be uploaded as a separate file during online submission labelled as File Type 'Abstract Figure'. Please also ensure that you include the figure legend in the main article file. All Abstract Figures should be created using BioRender. Authors should use The Journal's premium BioRender account to export high-resolution images. Details on how to use and access the premium account are included as part of this email.

- Your MS must include a complete "Additional information section" with the following 4 headings and content:

Competing Interests: A statement regarding competing interests. If there are no competing interests, a statement to this effect must be included. All authors should disclose any conflict of interest in accordance with journal policy.

Author contributions: Each author should take responsibility for a particular section of the study and have contributed to writing the paper. Acquisition of funding, administrative support or the collection of data alone does not justify authorship; these contributions to the study should be listed in the Acknowledgements. Additional information such as 'X and Y have contributed equally to this work' may be added as a footnote on the title page.

It must be stated that all authors approved the final version of the manuscript and that all persons designated as authors qualify for authorship, and all those who qualify for authorship are listed.

Funding: Authors must indicate all sources of funding, including grant numbers. If authors have not received funding, this must be stated.

It is the responsibility of authors funded by RCUK to adhere to their policy regarding funding sources and underlying research material. The policy requires funding information to be included within the acknowledgement section of a paper. Guidance on how to acknowledge funding information is provided by the Research Information Network. The policy also requires all research papers, if applicable, to include a statement on how any underlying research materials, such as data, samples or models, can be accessed. However, the policy does not require that the data must be made open. If there are considered to be good or compelling reasons to protect access to the data, for example commercial confidentiality or legitimate sensitivities around data derived from potentially identifiable human participants, these should be included in the statement.

Acknowledgements: Acknowledgements should be the minimum consistent with courtesy. The wording of acknowledgements of scientific assistance or advice must have been seen and approved by the persons concerned. This section should not include details of funding.

- Please upload separate high quality figure files via the submission form.

- Author profile(s) must be uploaded via the submission form. Authors should submit a short biography (no more than 100 words for one author or 150 words in total for two authors) and a portrait photograph of the two leading authors on the paper. These should be uploaded and clearly labelled together in a Word document with the revised version of the manuscript. Any standard image format for the photograph is acceptable, but the resolution should be at least 300 DPI and preferably more. A group photograph of all authors is also acceptable, providing the biography for the whole group does not exceed 150 words.

EDITOR COMMENTS

Reviewing Editor:

Thank you for submitting your well written and information review on fetal growth restriction and the associated fetal responses. It was generally well received by our reviewers but please address their concerns and suggestions for improvement. I tend to agree with Reviewer 2 that the evidence based for the front to back prioritisation is weaker than presented and should be tempered. The authors are ascribing teleologic meaning to the sequential changes in cerebral artery vasodilation: within this framework, can the authors explain the logic of why the most critical brain region i.e. the brainstem shows the slowest/last response? Is this because it's baseline bloodflow means it is protected while ACA and MCA vasodilation is occurring? If not it suggests it is being placed at risk while the fetus prioritises higher cortical areas - which would not make evolutionary sense. There is likely something simple here I have misunderstood but I'd appreciate the authors' insight.

Large portions of the summary sections are not referenced. While the authors speculations of course don't need references, there are multiple sentences that should be supported with references.

Finally although I take the authors point that 'brain sparing' is a bit misleading, I don't think anyone is truly misled and it is well appreciated that those fetuses with Doppler abnormalities are living close to the edge of placemtal function. In this section, the authors may want to use a different word that 'compromised' purely as this conjures thoughts of end stage fetal decompensation - which is not the authors intention.

REFEREE COMMENTS

Referee #1:

Please see attached review

Referee #2:

THE CAUSES AND CONSEQUENCES OF THE BRAIN SPARING RESPONSE IN FETAL GROWTH RESTRICTION

Allison B, White T, Camm E, Miller S.

This manuscript by Allison, White, Camm, and Miller provides a timely and comprehensive review of fetal brain sparing in the context of FGR, covering the clinical detection of brain sparing, the acute and chronic physiological mechanisms, and the postnatal neurological and cardiovascular consequences. The central conceptual contribution - a "front-to-back" hierarchy of cerebral arterial vasodilation evolving from compensatory to maladaptive - is well-articulated and provides useful clinical framing. The integration of preclinical large-animal data with human clinical Doppler studies is a strength. I have several major and minor concerns that I believe should be considered before the manuscript is accepted.

Major Issues

1. The novelty of this synthesis relative to prior reviews published in this journal and elsewhere (e.g., Giussani 2016, J Physiol; Miller et al. 2016, J Physiol) is not clearly established. The authors should explicitly articulate in the Introduction what conceptual framework or synthesis this review offers that was not available in those prior works.

2. The "front-to-back" hierarchy of cerebral vasodilation is presented as a relatively well-established sequential phenomenon (ACA → PCA/MCA → VA), but the clinical evidence base for this precise ordering is narrower than the manuscript implies. The primary evidence derives from a small number of studies, most prominently Figueroa-Diesel et al. 2007, and there is acknowledged variability across reports. The authors should temper the confidence with which this hierarchy is described,

and distinguish clearly between the sequence observed in severe early-onset FGR and what is known in the late-onset setting, where the evidence is considerably less robust.x

3. The transition from adaptive to maladaptive brain sparing is identified as a central unresolved question - appropriately so - but the manuscript does not adequately guide the reader toward what experimental approaches or mechanistic hypotheses are best positioned to resolve it. The review raises the question repeatedly but does not synthesize the existing evidence into testable predictions. A paragraph or subsection outlining the most tractable experimental gaps would substantially strengthen the review's contribution.

4. The cardiovascular programming section makes only a loose connection between brain sparing specifically and cardiovascular outcomes - the bulk of the evidence cited links FGR or low birth weight broadly to cardiovascular programming, not brain sparing as a distinct hemodynamic state. The authors should either strengthen this linkage (e.g., by focusing on studies that stratify cardiovascular outcomes by CPR or brain sparing status specifically) or acknowledge that the cardiovascular programming literature relates to FGR generally rather than the brain sparing response per se.

Minor Issues

1. Line 345: Typo - "Mmetabolic suppression" should read "Metabolic suppression."

2. Line 635: "it does it does not necessarily" should read "it does not necessarily."

END OF COMMENTS

THE CAUSES AND CONSEQUENCES OF THE BRAIN SPARING RESPONSE IN FETAL GROWTH RESTRICTION

Thank you for the opportunity to comment on the submitted document. This is a comprehensive and well-written review about the double-edged nature of the fetal brain sparing response shifting from initial protection to maladaptation associated with future risk of neurodevelopmental and cardiometabolic risks in the offspring. The summary illustration is wonderful, and my strong feeling is that the title of the review should be changed to ‘Fetal brain sparing: The double-edged sword of fetal hypoxaemia’. Maintaining the old review title will just lose this document in the literature and the readership will mistakenly assume that it describes most of what has already been published. The new title will avoid that mistake as it better reflects the novelty that this review brings. This will lead to broader readership and greater citation.

I have several comments below designed to increase the impact of the work that should be attended to:

Line 34. Change ‘Yet, the mechanisms that sustain brain sparing remain incompletely understood’ to ‘Yet, the mechanisms that maintain fetal brain sparing during sustained hypoxemia remain incompletely understood’

Line 62. Insert ‘(SGA) human infants’

Line 72. Please refrain from citing reviews that simply summarise the original discoveries and cite the original papers describing the seminal findings. In this case, please Remove Scherjon et al., 1993; Hernandez-Andrade et al., 2008; Giussani, 2016; Lear et al., 2024 and insert original reference that reported the discoveries PMID: 4429091 and PMID: 8350271

Line 113. Change ‘brain sparing is shown with head size relatively larger than body size, measured as an elevated ratio of head circumference or brain volume to abdominal circumference or liver volume’ to ‘brain sparing is shown with head size reduced less than overall body weight, measured as an elevated ratio of head circumference or brain volume to abdominal circumference or liver volume’

Line 115 change ‘large head…’ to ‘normal head diameter but shorter and/or thinner body size at birth’

Line 118. Remove sentence: As detailed below, this involves a significant degree of vasodilation of the cerebral blood vessels.

Lines 121-152. Please cite original articles rather than Tan and Roberts, 2021

Line 152 insert ‘increasing brain growth and cerebral vasodilation’

Line 222 vasodilator rather than vasodilatory

Lines 263-267 This statement is not quite correct, but it is not needed. Please delete lines 263-267.

Line 276 delete ‘as detailed below’

Line 285 add Giussani et al. 1993

Line 345 typo

Line 438 add PMID: 26926316

Line 444 The data reported in PMID: 11171645 show that adenosine is involved in the mechanisms mediating fetal cardiovascular, metabolic, and adrenergic responses to hypoxemia in fetal sheep. Please add a cited statement.

Line 445. Add PMID: 31884857

Line 465. Add PMID: 17588504

Line 586 Add PMID: 34694887

Line 588 Add PMID: 30668572

Line 591-608 The review has missed the recent work by Patey and colleagues (PMID: 40825560) showing that progressive fetal hypoxia in sheep leads to profound changes in fetal cardiac structure and function, resulting from a switch to LV dominance triggered by fetal brain sparing. The findings also indicate that fetal cardiac compensatory reserves become exhausted with progressive hypoxia. Many of these changes are observed in humans, as reported by Crispi et al, highlighting its translational relevance. Please include a statement to describe this relevant work.

Figure 3 would benefit from having citations on the left-hand side to support the statements made, which will increase the citation rate of this review. Such figures with citations are well acknowledged by the readership as they help target the early papers describing the original discoveries.

The summary figure is wonderful, but it shows the anatomy of an adult rather than a fetal heart. Please fix this.

Throughout please use the terms vasodilatation rather than vasodilation, and hypoxaemia rather than hypoxemia

Re: JP-TR-2026-291253 "**THE CAUSES AND CONSEQUENCES OF THE BRAIN SPARING RESPONSE IN FETAL GROWTH RESTRICTION**" by Beth J Allison, Tegan A White, Emily J Camm, and Suzanne L Miller

Please include an Abstract Figure file and an Abstract Figure legend.
Thank you, we have included both as required.

We sincerely thank the Editor and Reviewers for their careful evaluation of our manuscript and for their constructive and insightful feedback; in the revised, marked-up document, all changes from the original submission are highlighted in yellow.

EDITOR COMMENTS

Reviewing Editor:

I tend to agree with Reviewer 2 that the evidence based for the front to back prioritisation is weaker than presented and should be tempered.
We appreciate this feedback. We have tempered this discussion, now presenting the front-to-back vasodilatation as a working hypothesis and modifying the wording on the clinical findings that lend support for this directionality.

The authors are ascribing teleologic meaning to the sequential changes in cerebral artery vasodilation: within this framework, can the authors explain the logic of why the most critical brain region i.e. the brainstem shows the slowest/last response? Is this because it's baseline bloodflow means it is protected while ACA and MCA vasodilation is occurring? If not it suggests it is being placed at risk while the fetus prioritises higher cortical areas - which would not make evolutionary sense. There is likely something simple here I have misunderstood but I'd appreciate the authors' insight.
You raise an important point and we acknowledge that our original submission did not address it sufficiently. The clinical observations of an apparent front-to-back vasodilatation reflect only the cerebral artery response to hypoxaemia, however, many unknowns remain regarding regional physiological and cellular responses to low oxygen, including vasodilatory mechanisms and capacity, and cellular metabolic responses. The work of Morales Rosello et al 2012 (DOI: 10.1002/pd.3941) addresses this well, particularly from an evolutionary perspective. We have revised the wording to reflect that this area would benefit from further study.

Large portions of the summary sections are not referenced. While the authors speculations of course don't need references, there are multiple sentences that should be supported with references.
We have reviewed the summary sections and added supporting references to the statements that warrant them, while retaining unreferenced wording only where the text reflects our own speculation.

Finally although I take the authors point that 'brain sparing' is a bit misleading, I don't think anyone is truly misled and it is well appreciated that those fetuses with Doppler abnormalities are living close to the edge of placemtal function. In this section, the authors may want to use a different word that 'compromised' purely as this conjures thoughts of end stage fetal decompensation - which is not the authors intention.
We take the point that 'brain sparing', while imperfect, is unlikely to mislead readers. We agree, however, that 'compromised' may evoke end-stage fetal decompensation; this was not our intention and we have reworded this section accordingly (Line: 652-661).

REFEREE COMMENTS

Referee #2:

1. The novelty of this synthesis relative to prior reviews published in this journal and elsewhere (e.g., Giussani 2016, J Physiol; Miller et al. 2016, J Physiol) is not clearly established. The authors should explicitly articulate in the Introduction what conceptual framework or synthesis this review offers that was not available in those prior works.
Thank you for this helpful comment. We have revised the Introduction; we now explicitly state our intended scope for this review.

2. The "front-to-back" hierarchy of cerebral vasodilation is presented as a relatively well-established sequential phenomenon (ACA → PCA/MCA → VA), but the clinical evidence base for this precise ordering is narrower than the manuscript implies. The primary evidence derives from a small number of studies, most prominently Figueroa-Diesel et al. 2007, and there is acknowledged variability across reports. The authors should temper the confidence with which this hierarchy is described, and distinguish clearly between the sequence observed in severe early-onset FGR and what is known in the late-onset setting, where the evidence is considerably less robust.
We appreciate this point raised. Only three clinical papers have assessed multiple cerebral arteries in growth restricted fetuses, and while they all broadly demonstrate a dynamic cerebrovascular pattern, the front-to-back hierarchy requires further substantiation. We have revised the wording in a number of sections to present the front-to-back pattern of vasodilatation as a working hypothesis that requires further validation, and to distinguish more clearly between the sequence observed in severe early-onset FGR and the considerably less robust evidence in the late-onset setting.

3. The transition from adaptive to maladaptive brain sparing is identified as a central unresolved question - appropriately so - but the manuscript does not adequately guide the reader toward what experimental approaches or mechanistic hypotheses are best positioned to resolve it. The review raises the question repeatedly but does not synthesize the existing evidence into testable predictions. A paragraph or subsection outlining the most tractable experimental gaps would substantially strengthen the review's contribution.
We thank the reviewer for this excellent suggestion. The final section of the review is now titled ‘Summary and Future Directions’ and includes a paragraph outlining the studies best positioned to add critical insight and real-time information for characterising prolonged brain sparing.

4. The cardiovascular programming section makes only a loose connection between brain sparing specifically and cardiovascular outcomes - the bulk of the evidence cited links FGR or low birth weight broadly to cardiovascular programming, not brain sparing as a distinct hemodynamic state. The authors should either strengthen this linkage (e.g., by focusing on studies that stratify cardiovascular outcomes by CPR or brain sparing status specifically) or acknowledge that the cardiovascular programming literature relates to FGR generally rather than the brain sparing response per se.
We thank the reviewer for this constructive critique. We agree that the original text did not clearly distinguish between cardiovascular programming linked to FGR broadly from that specifically attributable to the brain sparing haemodynamic state. We have revised the cardiovascular programming section accordingly. In the revised text, we now explicitly acknowledge that CPR-stratified cardiovascular outcome data are limited and that much of the existing literature characterises cardiovascular risk as a function of FGR severity rather than brain sparing status *per se*. Where the evidence allows, we have also strengthened

the mechanistic and evidential linkage to brain sparing as a distinct haemodynamic exposure.

Minor Issues

1. Line 345: Typo - "Mmetabolic suppression" should read "Metabolic suppression."
Thank you, amended.

2. Line 635: "it does it does not necessarily" should read "it does not necessarily."
Thank you, amended.

Reviewer #1

My strong feeling is that the title of the review should be changed to ‘Fetal brain sparing: The double-edged sword of fetal hypoxaemia’. Maintaining the old review title will just lose this document in the literature and the readership will mistakenly assume that
it describes most of what has already been published. The new title will avoid that mistake as it better reflects the novelty that this review brings. This will lead to broader readership and greater citation.
Thank you for the suggestion. We have amended the title. The new title is ‘Brain Sparing in Fetal Growth Restriction: The Double-Edged Sword of Fetal Hypoxaemia’.

Line 34. Change ‘Yet, the mechanisms that sustain brain sparing remain incompletely understood’ to ‘Yet, the mechanisms that maintain fetal brain sparing during sustained hypoxemia remain incompletely understood’
Thank you, this has been amended.

Line 62. Insert ‘(SGA) human infants’
Thank you, this has been amended.

Line 72. Please refrain from citing reviews that simply summarise the original discoveries and cite the original papers describing the seminal findings. In this case, please Remove Scherjon et al., 1993; Hernandez-Andrade et al., 2008; Giussani, 2016; Lear et al., 2024 and insert original reference that reported the discoveries PMID: 4429091 and PMID: 8350271.
Thank you. This is a very important point, and it has been amended.

Line 113. Change ‘brain sparing is shown with head size relatively larger than body size, measured as an elevated ratio of head circumference or brain volume to abdominal circumference or liver volume’ to ‘brain sparing is shown with head size reduced less than overall body weight, measured as an elevated ratio of head circumference or brain volume to abdominal circumference or liver volume’. Amended.

Line 115 change ‘large head…’ to ‘normal head diameter but shorter and/or thinner body size at birth’. Amended.

Line 118. Remove sentence: As detailed below, this involves a significant degree of vasodilation of the cerebral blood vessels.
Amended.

Lines 121-152. Please cite original articles rather than Tan and Roberts (2021).
We had added original articles to this paragraph.

Line 152 insert ‘increasing brain growth and cerebral vasodilation’.
Amended.

Line 222 vasodilator rather than vasodilatory.
Amended.

Lines 263-267 This statement is not quite correct, but it is not needed. Please delete lines263-267.
Removed.

Line 276 delete ‘as detailed below’.
Amended.

Line 285 add Giussani et al. 1993,
Added.

Line 345 typo.
Amended.

Line 438 add PMID: 26926316.
Added.

Line 444 The data reported in PMID: 11171645 show that adenosine is involved in the mechanisms mediating fetal cardiovascular, metabolic, and adrenergic responses to hypoxemia in fetal sheep. Please add a cited statement.
Added.

Line 445. Add PMID: 31884857.
Added.

Line 465. Add PMID: 17588504.
Added.

Line 586 Add PMID: 34694887.
Added.

Line 588 Add PMID: 30668572.
Added.

Line 591-608 The review has missed the recent work by Patey and colleagues (PMID: 40825560) showing that progressive fetal hypoxia in sheep leads to profound changes in fetal cardiac structure and function, resulting from a switch to LV dominance triggered by fetal brain sparing. The findings also indicate that fetal cardiac compensatory reserves become exhausted with progressive hypoxia. Many of these changes are observed in humans, as reported by Crispi et al, highlighting its translational relevance. Please include a statement to describe this relevant work.
We apologise for this oversight. This manuscript has now been included.

Figure 3 would benefit from having citations on the left-hand side to support the statements made, which will increase the citation rate of this review. Such figures with citations are well acknowledged by the readership as they help target the early papers describing the original discoveries.
Citations added.

The summary figure is wonderful, but it shows the anatomy of an adult rather than a fetal heart. Please fix this.
Amended.

Throughout please use the terms vasodilatation rather than vasodilation, and hypoxaemia rather than hypoxemia.
Amended.

Dear Professor Miller,

Re: JP-TR-2026-291253R1 "**BRAIN SPARING IN FETAL GROWTH RESTRICTION: THE DOUBLE-EDGED SWORD OF FETAL HYPOXAEMIA**" by Beth J Allison, Tegan A White, Emily J Camm, and Suzanne L Miller

We are pleased to tell you that your paper has been accepted for publication in The Journal of Physiology.

PLEASE NOTE- Please include an Abstract Figure legend. An appropriate figure legend, which should not exceed 150 words in length, should be included in the main manuscript file.

TRANSPARENT PEER REVIEW POLICY: To improve the transparency of its peer review process, The Journal of Physiology publishes online as supporting information the peer review history of all articles accepted for publication. Readers will have access to decision letters, including Editors' comments and referee reports, for each version of the manuscript, as well as any author responses to peer review comments. Referees can decide whether or not they wish to be named on the peer review history document.

The last Word (or similar) version of the manuscript provided will be used by the Production Editor to prepare your proof. When this is ready you will receive an email containing a link to Wiley's Online Proofing System. The proof should be thoroughly checked and corrected as promptly as possible.

Authors should note that it is too late at this point to offer corrections prior to proofing. Major corrections at proof stage, such as changes to figures, will be referred to the Editors for approval before they can be incorporated. Only minor changes, such as to style and consistency, should be made at proof stage. Changes that need to be made after proof stage will usually require a formal correction notice.

All queries at proof stage should be sent to: TJP@wiley.com.

Are you on Twitter? Once your paper is online, why not share your achievement with your followers? Please tag The Journal (@jphysiol) in any tweets and we will share your accepted paper with our 30,000 followers!

Yours sincerely,

Laura Bennet
Senior Editor
The Journal of Physiology

P.S. - You can help your research get the attention it deserves! Check out Wiley's free Promotion Guide for best-practice recommendations for promoting your work at www.wileyauthors.com/eeo/guide. You can learn more about Wiley Editing Services which offers professional video, design, and writing services to create shareable video abstracts, infographics, conference posters, lay summaries, and research news stories for your research at www.wileyauthors.com/eeo/promotion.

• IMPORTANT NOTICE ABOUT OPEN ACCESS: To assist authors whose funding agencies mandate immediate public access to published research findings, The Journal of Physiology allows authors to pay an Open Access (OA) fee to have their papers made freely available immediately on publication.

The Corresponding Author will receive an email from Wiley with details on how to register or log in to Wiley Authors where you will be able to place an order.

You can check if your funder or institution has a Wiley Open Access Account here:
https://authors.wiley.com/author-resources/Journal-Authors/open-access/author-compliance-tool.html

----

EDITOR COMMENTS

Reviewing Editor:

Thank you for your careful revisions. Congratulations on an excellent review and thank you for your contribution to The Journal.

REFEREE COMMENTS

Referee #1:

All items addressed satisfactorily in the amended version.

Referee #2:

In my opinion, the authors have done a nice job replying to the issues raised by the editor and reviewers. I have no further concerns, and feel that this manuscript is a valuable addition to the field that will help keep basic researchers focused on the clinical challenges.

****
